# Complex duodenal fistulae: a surgical nightmare

**DOI:** 10.1186/s13017-023-00503-w

**Published:** 2023-05-19

**Authors:** Ari Leppäniemi, Matti Tolonen, Panu Mentula

**Affiliations:** 1 Abdominal Center, Division of Emergency Surgery, Helsinki University Hospital, University of Helsinki, Helsinki, Finland; 2 Meilahti Tower Hospital, Haartmaninkatu 4, 00029 Helsinki, Finland

**Keywords:** Duodenal leak, Duodenal fistula, Enteric fistula, Postoperative peritonitis, Intra-abdominal infection, Duodenal diversion

## Abstract

**Introduction:**

A common feature of external duodenal fistulae is the devastating effect of the duodenal content rich in bile and pancreatic juice on nearby tissues with therapy-resistant local and systemic complications. This study analyzes the results of different management options with emphasis on successful fistula closure rates.

**Methods:**

A retrospective single academic center study of adult patients treated for complex duodenal fistulas over a 17-year period with descriptive and univariate analyses was performed.

**Results:**

Fifty patients were identified. First line treatment was surgical in 38 (76%) cases and consisted of resuture or resection with anastomosis combined with duodenal decompression and periduodenal drainage in 36 cases, rectus muscle patch, and surgical decompression with T-tube in one each. Fistula closure rate was 29/38 (76%). In 12 cases, the initial management was nonoperative with or without percutaneous drainage. The fistula was closed without surgery in 5/6 patients (1 patient died with persistent fistula). Among the remaining 6 patients eventually operated, fistula closure was achieved in 4 cases. There was no difference in successful fistula closure rates among initially operatively versus nonoperatively managed patients (29/38 vs. 9/12, *p* = 1.000). However, when considering eventually failed nonoperative management in 7/12 patients, there was a significant difference in the fistula closure rate (29/38 vs. 5/12, *p* = 0.036). The overall in-hospital mortality rate was 20/50 (40%).

**Conclusions:**

Surgical closure combined with duodenal decompression in complex duodenal leaks offers the best chance of successful outcome. In selected cases, nonoperative management can be tried, accepting that some patients may require surgery later.

## Introduction

Duodenal leaks are most seen postoperatively in duodenal suture or anastomotic lines following duodenal repair or resection, or in some cases following persistent external duodenal fistulae after percutaneous management of iatrogenic duodenal lesions. The leak can be contained in the retroperitoneum but is usually intraperitoneal. The common feature of this type of duodenal leak is the effect of the duodenal content rich in bile and pancreatic juice on nearby tissues, hence called “complex” duodenal leaks for the purpose of this analysis.

While early repair of a healthy duodenal perforation is straight-forward and usually healing well, the approach to an edematous, friable duodenum soaked in bile for a few days is very different as suggested by the abundant literature and techniques usually involving just a small number of patients [1–15] (Table 1). Obviously, there are now randomized studies to suggest the best treatment strategy, and perhaps there is no one technique that fits all [16].

**Table 1:** Management techniques of duodenal leaks and external duodenal fistulas

Technique	1st author	Year	N	Success rate
Serosal patch	Kittrick	1965	1	1/1
Initial nonoperative and drainage	Garden	1988	24	22/24 (92%)
Transparietal endoscopic intubation	Bloch	1989	2	2/2
Initial nonoperative	Williams	1997	13	8/13 (62%)
Serosal patch	Curti	1997	4	4/4
Intubation and intraluminal irrigation	Parc	1999	49	32/49 (65%)
Gastrostomy tube fistula plug	Halttunen	2003	2	2/2
Rectus abdominis muscle flap	Chander	2004	6	6/6
Percutaneous transhepatic duodenal diversion	Zarzour	2008	6	5/6
Biliogastric diversion	Milias	2009	2	2/2
T-tube duodenocholangiostomy	Paluszkiewics	2010	12	12/12
Modified duodenal diverticulization	Cruz	2010	3	3/3
T-tube decompression	Gupta	2013	10	10/10
Percutaneous sinus tract drainage	Shen	2019	10	9/10
Prophylactic modified tube	Jung	2020	5	4/5

The aim of this study was to describe the different management options with emphasis on successful fistula closure rates and to identify principles that could be used alone or in combination to manage these challenging patients.

## Methods

This study was conducted as a retrospective cohort study from a single academic center that serves both as a secondary and a tertiary referral hospital. Institutional review board approved the study design. Clinical data were collected from electronic hospital records over a period from April 1, 2004, until March 31, 2021. The period starts with the implementation of a new electronic hospital patient record system.

A complex duodenal leak was defined as a full-thickness intra- or retroperitoneal perforation of the duodenal suture or anastomotic line following duodenal repair. In addition, post-endoscopy duodenal perforations initially managed nonoperatively with percutaneous drains were included. Patients with gastric and post-duodenal enteral leaks or fistulas, peptic, post-traumatic, iatrogenic or other duodenal perforations managed successfully at the initial operation, internal duodenal fistulas (such as cholecystoduodenal, or aortoduodenal fistulas), and post-endoscopy perforations managed without percutaneous or surgical interventions were excluded.

Comorbidities were classified according to Charlson Comorbidity Index (CCI) [17], and the ASA class according to the American Society of Anesthesiologists (ASA) Physical Status Classification [18]. Sepsis was classified according to the sepsis-3 criteria [19].

### Statistical analyses

Patient characteristics and perioperative data are presented in number and percentage of patients, mean and median, range and interquartile range (IQR), where appropriate. Univariate analyses for categorical variables were performed using Fischer´s exact test or Chi-Square test and for continuous variables Mann–Whitney U test, or Student *t* test, where appropriate. Normality of continuous variables distribution was analyzed with Shapiro–Wilk test. Two-tailed *p* < 0.05 was considered significant. All patients’ data were analyzed more than 30 days postoperatively. Analyses were performed using SPSS© Statistics version 25 for Mac (IBM©, Armonk, NY, USA).

## Results

### Clinical characteristics

Out of some 2500 adult patients undergoing surgical or endoscopic interventions to the duodenum during the 17-year period, 72 patients with potential duodenal fistulas (ICU admission, repeated surgical, endoscopic, or percutaneous duodenal interventions, prolonged hospital stay) were identified. After excluding 22 patients who eventually did not have a duodenal leak, 50 adult patients with complex duodenal leaks as defined above were identified and form the study population.

Clinical characteristics of the patients and duodenal leaks are summarized in Tables 2 and 3. The median (IQR) delay from the initial intervention to the diagnosis of the leak was 4 (2–8.5) days, and the delay from diagnosis to the first attempt to treat the leak 0 (0–0.25) days.

**Table 2:** Clinical characteristics of the 50 patients with duodenal leaks

	*n*	%
Male gender	36	72
Age (years, median, range)	63.5	28–86
Charlson comorbidity index (median, range)	2	0–6
Sepsis/septic shock	10/3	20/6
*Underlying disease or condition*		
Peptic ulcer perforation	16	32
Severe acute pancreatitis	11	22
Iatrogenic or traumatic perforation	11	22
Gastric malignancy	6	12
Infected aortic prosthesis	4	8
Gallbladder malignancy	1	2
Benign duodenal stricture	1	2
*Primary duodenal intervention*		
Duodenal suture	27	54
Duodenal resection with anastomosis	16	32
ERCP*	3	6
Transduodenal drainage with PEG**	2	4
Omental plug	1	2
None***	1	2

*ERCP, endoscopic retrograde cholangio-pancreatography, **PEG, percutaneous endoscopic gastrostomy, ***duodenal perforation was diagnosed from bile leak in the drain after necrosectomy for infected pancreatic necrosis

**Table 3:** Clinical characteristics of the duodenal leaks

	*n*	%
*Principal diagnostic method for the duodenal leak*		
Bile-stained drainage from periduodenal drain	27	54
Found at reoperation	10	20
Positive methylene blue test	6	12
Imaging	5	10
Bile-stained drainage from wound	2	4
*Location of the duodenal leak*		
Intraperitoneal	45	90
Retroperitoneal	5	10
Part 1 of the duodenum	26	52
Part 2 of the duodenum	18	36
Part 3 of the duodenum	6	12
*Type of the duodenal leak*		
Suture line dehiscence	24	48
Duodenal stump	11	22
Duodenal anastomotic dehiscence	7	14
Associated with acute pancreatitis	4	8
After ERCP* or removal of a duodenal stent	4	8

*ERCP, endoscopic retrograde cholangio-pancreatography

### Management

The initial management strategy of the duodenal leak was operative in 38 (76%) patients and nonoperative in 12 (24%). Nonoperative management consisted of percutaneous (and endoscopically assisted as needed) drainage with repeated change or shortening of the drain.

The procedures performed for the 38 initially operatively managed patients are listed in Table 4. In addition to the attempted closure of the leak, one or a combination of methods were used to protect the suture or anastomotic line by decompressing the duodenum. One or more external periduodenal drains were used in all operatively managed patients and a feeding jejunostomy in 63%. The fistula closure rate was 29/38 (76%).

**Table 4:** Surgical procedures performed in 38 patients undergoing initial operative management of a duodenal leak

	*n*	%
Suture closure of the duodenal leak	30	79
Resection with duodenal or duodenojejunal anastomosis	6	16
Rectus muscle patch only	1	3
Duodenal decompression (and T-tube) only	1	3
*Additional decompression methods*		
Nasogastroduodenal tube	16	42
Retrograde duodenal tube	17	45
Biliary diversion (T-tube or PTC*)	16	42
Nasogastric tube	34	89
*Additional procedures*		
Periduodenal external drain	38	100
Feeding jejunostomy	24	63

*PTC, percutaneous transhepatic cholangiography

In 12 cases, the initial management was nonoperative with or without percutaneous drainage. The fistula was closed without surgery in 5/6 patients (1 patient died with persistent fistula) within 6–29 (mean 15) days and verified with oral contrast CT scan and/or clearing of the periduodenal drain secretion. Among the remaining 6 patients, eventually 3 required duodenal resection or repair and 3 operative drainage (with T-tube in one), and fistula closure was achieved in 4/6 cases.

### Fistula closure rates and outcomes

Overall, while the closure of the duodenal leak was successful in 38 patients (76%), eventually 30 patients survived. Of the 8 patients who died 11–36 (mean 22) days after the management of the fistula all but two (one suffered an acute myocardial infarct, and another was a multiple sclerosis patient who died of respiratory insufficiency) died of multiple organ failure that can in most cases be attributed to the leak and associated sepsis. The success rates and survival rates in each management category are summarized in Table 5. There was no difference in successful fistula closure rates among initially operatively versus nonoperatively managed patients (29/38 vs. 9/12, *p* = 1.000). However, when considering eventually failed nonoperative management in 7/12 patients, there was a significant difference in the fistula closure rate (29/38 vs. 5/12, *p* = 0.036). Furthermore, the primary intervention was more successful in patients with peptic ulcer perforation compared with other initial diagnoses (12/16 vs. 15/34, *p* = 0.041) or when the surgeon performing the initial intervention was a full-time emergency surgeon vs. elective surgeon (19/28 vs. 8/22, *p* = 0.027).

**Table 5:** Fistula closure and survival rates after different management strategies

	Successful fistula closure	Survival rate
*Initial operative management*		
Suture closure	22/30 (73%)	17/30 (57%)
Resection with anastomosis	6/6	4/6 (67%)
Patch only	0/1	0/1
Decompression only	1/1	1/1
Subtotal	29/38 (76%)	22/38 (58%)
*Initial nonoperative management*		
NOM* only	5/6 (83%)	5/6 (83%)
NOM* + subsequent surgery	4/6 (67%)	3/6 (50%)
Subtotal	9/12 (75%)	8/12 (67%)
Total	38/50 (76%)	30/50 (60)

*NOM, nonoperative management

Out of the total of 50 patients, 20 patients (40%) died, 16/38 (42%) of those initially managed operatively, and 4/12 (33%) of those undergoing initial nonoperative management (Table 5). The causes of death included multiple organ failure (MOF) in 11 patients, persistent infection in 6, respiratory failure in 2 and acute myocardial infarction in one patient. Forty-three (86%) patients required management in the ICU during the fistula management for a median of 12 (IQR 4–32, range 1–143) days. The overall median length of hospital stays including readmissions for the survivors was 12 (IQR 4–32, range 1–43) days.

## Discussion

The main findings in this study showed that the majority of patients with duodenal fistulae can be successfully managed with surgical intervention consisting of duodenal repair or resection, especially when performed by a surgeon with experience in emergency surgery. In selected cases, nonoperative management with or without percutaneous drainage was successful even though some patients required surgery later.

Duodenal fistulas are rare and occur in 2–7% after repair of perforated peptic ulcer, in about 4% in associated with severe acute pancreatitis, 3% after gastrectomy for gastric cancer and 1% after endoscopic retrograde cholangio-pancreatography (ERCP) [20–23]. The main controversy regarding the management of postoperative duodenal fistulas is between initial surgical or nonoperative management, and as shown in Table 1, the successful fistula closure rates in series with more than 10 patients vary from 62 to 100%.

In a series of 24 patients with postoperative duodenal fistulas, management consisted of aggressive nutritional support, localization and drainage of intra-abdominal sepsis, and definitive surgical closure for those fistulas which did not close spontaneously. Spontaneous closure occurred in 22 (92%) cases; however, 14 patients had a total of 19 operations for drainage of localized wound or intra-abdominal abscess. The remaining two patients subsequently underwent definitive surgical closure at five and six weeks. The mortality rate was 8% [2].

Parc et al. reported 49 cases with postoperative peritonitis originating from a duodenal leak. The surgical management consisted of insertion of a spiral drain into the duodenum through the leaking site with its intraluminal end directed distally, external drainage and feeding jejunostomy. Infusion of 2000 ml/24 h with normal saline (containing thrombin, tranexamic acid and rifampicin) was started at the end of the operation and was continued for a mean of 21 days after which the spiral drain was removed and replaced with a 12-French silicone drain which was progressively removed to permit the closure of the fistula. In 32 (65%) patients, the duodenal fistula closed spontaneously at a median time of 39 (range 19–120) days. The overall mortality rate was 22% [6].

In the present series, the majority of patients (38/50, 76%) underwent initial surgical management and in all but one there was an attempt to close the duodenal defect by suture (22 patients), resection and anastomosis (6) or rectus muscle patch (one), resulting in successful fistula closure in 28/37 (76%) cases (Table 5). Specifically, in all 6 patients undergoing resection and anastomosis, the fistula remained closed.

One key element in successful repair seems to be the protection of the suture or anastomotic line with adequate duodenal decompression, either via antegrade naso-gastro-duodenal or retrograde duodenal tube, and sometimes augmented with cholecystectomy and insertion of a T-tube to achieve biliary diversion (Table 4). Duodenal decompression can also be achieved with a tube duodenostomy. It was used in a series of 31 patients with potentially insecure duodenal stump closure (12 patients) or postoperative duodenal leakage (12) through the open end of duodenum and augmented with a T-tube for biliary diversion in 19 patients. Only one patient (3%) had a subsequent duodenal stump leak which healed spontaneously [24].

Protection of the duodenal suture line by pyloric exclusion in the management of duodenal fistulae was reported already in 1907 and has subsequently been used in the management of traumatic duodenal perforations, although its benefit in traumatic setting has not been established [25–27]. In extreme situations, a duodenal diverticulization procedure (gastric antrectomy, tube duodenostomy, gastrojejunostomy, external periduodenal drainage and insertion of a T-tube for biliary drainage) has been used for extensive duodenal injuries [28] and was successfully used in one case in the present series.

Our current practice includes double-decompression; a nasogastric tube to the stomach and a naso-gastro-duodenal tube with extra side holes to the duodenum. In selected cases, we add a T-tube to facilitate the decompressive effect. Inserting a periduodenal external drain is mandatory and can sometimes control a secondary leak and help to avoid a reoperation. Enteral nutrition via feeding jejunostomy or in cases of a Roux-en-Y reconstruction via nasojejunal tube should be started as soon as possible. Although we used tube duodenostomy for decompression in past, we currently avoid making extra holes to the duodenum and replace tube duodenostomy with intraluminal decompression methods.

In selected cases, nonoperative management often combined with percutaneous drainage can be successful. In a systematic review of duodenal stump fistulae after gastrectomy for gastric cancer, conservative approach was performed in 79 stable patients with complete resolution achieved in 92% with a healing time ranging from 17 to 71 days [29]. Among 29 patients with external duodenal fistulae following closure of duodenal perforation and surviving 48 h, 14 patients (48%) were initially managed nonoperatively, out of which six required later surgery. In 15 patients, the indications for early surgery were peritonitis or failure to establish enteral feeding. The fistula closed spontaneously in 8/14 patients managed conservatively within 9–58 (mean 28) days, 2/6 patients with delayed surgery died. The mortality rate after initial operated patients was 9/15 (60%), but the successful closure rate was not reported [30].

Of the 12 patients undergoing initial nonoperative management in the present series, 5 were managed successfully with one or more percutaneous or endoscopic interventions, and one patient died of MOF and persistent fistula. Of the 6 patients with failed nonoperative management requiring subsequent surgery, the fistula remained closed in 4 patients, out of which one died later of sepsis caused by therapy-resistant intra-abdominal abscesses while the duodenum remained intact. In the remaining two cases, the fistula could not be closed despite surgery, and both patients died of MOF. It seems that in selected cases of stable patients and with no generalized peritonitis, nonoperative management including percutaneous and endoscopic drainage procedures can be attempted, but if failing to control the fistula, prompt operative intervention might rescue some of the patients.

In a literature review from 1865 to 1937, Bartlett and Holwell reported 130 cases of postoperative duodenal fistulae and added 12 cases of their own. It included two reports with 61 and 44 cases, where the mortality rates were 51% and 18%, respectively [31]. In more recent series, the mortality rate has varied between 8 and 42% [2, 4, 6, 22, 24, 30].

The overall 40% mortality rate in this series reflects the severity and the challenges facing surgeons treating these patients. Although some of the mortality can partly be associated with the underlying disease such as severe acute pancreatitis, obviously the duodenal leak and associated sepsis can be considered a significant contributing factor in all cases. Due to the small number of patients, no single independent risk factor for mortality could be identified.

The limitations of this study are related to its retrospective nature, small and heterogenous study population, and wide range of the management strategy.

## Conclusions

External duodenal fistulae are rare but when occurring, pose a significant surgical challenge. Intraluminal duodenal decompression at the initial surgical intervention involving the duodenum could potentially decrease the risk of leakage, and the placement of a periduodenal drain facilitate early diagnosis. Prompt surgery is often the best option before the deleterious effects of the duodenal content to the tissues takes its full course. Surgical closure with suture or resection and anastomosis should be attempted and combined with adequate intraluminal decompression and sometimes biliary diversion with a T-tube. In patients where an initial suture line has dehisced, resection might be a better alternative. Adequate nutritional support preferably via enteral route and appropriate antimicrobial treatment are important. In selected cases of stable patients and with no generalized peritonitis, nonoperative management including percutaneous and endoscopic drainage procedures can be attempted, but if failing to control the fistula, operative intervention is required.

## Data Availability

Not applicable.

## Abbreviations

ASA: American Society of Anesthesiologists
CCI: Charlson comorbidity index
CT: Computed tomography
ERCP: Endoscopic retrograde cholangio-pancreatography
ICU: Intensive care unit
IQR: Interquartile range
MOF: Multiple organ failure
NOM: Nonoperative management
PEG: Percutaneous endoscopic gastrostomy
PTC: Percutaneous transhepatic cholangiography
